# New Nutrient Rich Food Nutrient Density Models That Include Nutrients and MyPlate Food Groups

**DOI:** 10.3389/fnut.2020.00107

**Published:** 2020-07-21

**Authors:** Adam Drewnowski, Victor L. Fulgoni

**Affiliations:** ^1^Center for Public Health Nutrition, University of Washington, Seattle, WA, United States; ^2^Nutrition Impact LLC, Battle Creek, MI, United States

**Keywords:** nutrient density, nutrient-rich food index, Healthy Eating Index 2015, nutrients, food groups, nutrient profiling, My Plate

## Abstract

**Background:** Dietary guidelines have shifted emphasis from single nutrients to food patterns, food groups, and dietary ingredients. Nutrient profiling models need to do the same.

**Methods:** Dietary intake data for 23,643 persons aged >2 years came from the 2011–2016 National Health and Examination Survey (NHANES 2011-16). Healthy Eating Index HEI-2015 was the diet quality measure. The new Nutrient Rich Food hybrid score (NRFh) was based on three subscores. The subscore based on x nutrients to encourage was defined as NRx. The subscore based on y MyPlate food groups to encourage was MPy. The negative subscore based on z nutrients to limit was LIMz. The final algorithm was NRFh(x.y.z) = NRx + MPy − LIMz. The selection of NRFh model components from among 16 nutrients and five food groups was based on regression analyses.

**Results:** We conducted a total of 2,162,720 iterative regression analyses against HEI-2015 diet quality scores. NRF scores based on 16 nutrients accounted for up to 66% of the variance, whereas scores based on 5 MP food groups accounted for 50%. The new NRFh3:4:3 score with six nutrients and four food groups (fiber, potassium, PUFA+MUFA; whole grains, dairy, fruit, nuts and seeds; saturated fat, added sugar, sodium) explained 72%. The new NRFh4:3:3 score with seven nutrients and three food groups (protein, fiber, potassium, PUFA+MUFA; whole grain, dairy, fruit; saturated fat, added sugar, sodium;) also explained 72%. In both NRFh models, regressions remained significant for each population subgroup examined.

**Conclusion:** The NRFh3:4:3 and NRFh4:3:3 models correlated well with HEI-2015 scores, a measure of diet quality that tracks compliance with Dietary Guidelines. Hybrid NP models based on nutrients and food groups could become part of dietary guidance.

## Introduction

The Dietary Guidelines for Americans (DGA), reissued every 5 years, are the basis of food and nutrition policy in the US (1–8). The DGA have continuously stressed the importance of nutrient-rich food patterns that meet the recommendations for essential nutrients (protein, fiber, vitamins, and minerals) while limiting intakes of saturated fat, added sugar, and sodium (8). The 2005 DGA introduced the concept of nutrient density, advising Americans to limit discretionary calories and consume a wider variety of nutrient-rich foods (6).

Since then, quantitative methods to assess the nutrient density of foods have become known as nutrient profiling (9, 10). The goal of nutrient profiling (NP) models is to identify nutrient-rich foods and separate them from foods of lower nutritional value (11). This is done by calculating each food's nutrient content per 100 g, 100 kcal, or per serving (12). NP models have provided the scientific basis for front-of-pack labels (13–15), health and nutrition claims (16), and regulating marketing and advertising to children (17, 18). NP models have also become the scientific basis for product reformulation by the food industry (19–21).

The 1980 DGA had already featured the advice to eat a wider variety of foods (1). More recent issues of the DGA have emphasized healthy food patterns even more (8). A healthy eating pattern includes whole grains, low-fat dairy, vegetables and fruit, and a variety of protein foods such as seafood, lean meats and poultry, eggs, legumes, and nuts, seeds, and soy, along with healthy oils (8, 22). Whereas, the DGA have become more food oriented, most NP models have remained purely nutrient-based (9, 10, 22, 23). Although some NP models do award an arbitrary number of points for the food's content of fruits, vegetables, and nuts (FVN), their overall scores tend to be driven by energy density and by the amounts of added sugars and fat (18).

A case can be made for an alternative hybrid approach to NP, one that would integrate selected nutrients with My Plate food groups (24, 25). One recent study (26) pointed to the value of the ingredient list as a potential source of data for nutrient profiling. When listed in the first place, dairy and fruit were associated with higher NRF8.3 nutrient density scores (26).

The present approach used multiple regression analyses to create and test new Nutrient Rich Food hybrid nutrient density score(s) that would include both nutrients and desirable My Plate food groups. Multiple regression analyses were conducted to determine which combination of nutrients and food groups would be best aligned with the Healthy Eating Index (HEI-2015) diet quality scores.

The HEI-2105, used here for validation purposes, is a measure of diet quality that is based on adherence to the DGA (27–29). The HEI-21015 includes multiple adequacy and moderation components, most of which are expressed as densities, relative to calories (27). An updated HEI is released to accompany each new edition of the DGA (28) and can be used to monitor changes in dietary patterns following interventions. Studies in multi-ethnic populations suggest that following a diet consistent with the DGA and characterized by higher HEI-2015 scores was associated with reduced mortality from CVD, cancer, and all-cause (29).

## Methods

### NHANES 2011-16 Participants

Dietary intake data came from three cycles of the nationally representative National Health and Nutrition Examination Survey for years 2011–2016 (NHANES 2011-16). Analyses were performed on data for the first day of intake for all persons >2 years old, with exclusions for incomplete data, pregnant or lactating females, or energy intakes equal to zero. There were 27,925 participants >2 years, of whom 4,007 had incomplete data, 298 were pregnant or lactating females, and 2 had kcal = 0. The final analytical sample was 23,743.

The NHANES participants were stratified by gender and age. The age group cut-points were: 2–18, 19–50, and >50 years. These age groups generally correspond to the age groups used by the Institute of Medicine to examine Dietary Reference Intakes. Race/ethnicity was defined as: non-Hispanic white; non-Hispanic black, Hispanic and Asian. Family income-to-poverty ratio (IPR) is the ratio of family income to the federal poverty threshold; the cut-points for IPR were ≤ 1.85 or >1.85. Education was coded as high school or less; completed high school; some college; and completed college.

Obesity in adults was defined as BMI>30 based on measured heights/weights (body mass index = kg/m^2^). Overweight was BMI ≥25 to <30), and normal weight was BMI <25. Obesity in children was defined as BMI for age ≥95th percentile, overweight as BMI for age ≥85th and >95th percentile, and normal weight as BMI for age <85th percentile.

### IRB and Ethical Approvals

The necessary IRB approval for NHANES had been obtained by the National Center for Health Statistics (NCHS) (30). Adult participants provided written informed consent. Parental/guardian written informed consent was obtained for children. Children/adolescents ≥12 years provided additional written consent. All NHANES data are publicly available on the NCHS and USDA websites (31). All documentation of laboratory methodology, including plasma lipid analyses, is provided online at wwwn.cdc.gov. Per University of Washington (UW) policies, public data do not involve “human subjects” and require neither additional IRB review nor an exempt determination. Such data may be used without any involvement of the Human Subjects Division or the UW Institutional Review Board.

### The Healthy Eating Index (HEI 2015) Diet Quality Measure

The USDA Food and Nutrient Database for Dietary Studies (FNDDS) was used to calculate the energy and nutrient content of the NHANES diet (32). The Food Patterns Equivalents Database (FPED) from the United States Department of Agriculture (USDA) was used to estimate the intakes of food groups of interest and to calculate HEI 2015 scores (33).

The HEI-2015 was specifically designed to monitor compliance with the 2015 Dietary Guidelines for Americans (32). The HEI-2015 is an energy adjusted summary measure of diet quality based on the intake of 9 food groups/nutrients to encourage: total fruits, whole fruits, total vegetables, greens and beans, whole grains, dairy, total protein foods, seafood and plant protein, and fatty acids ratio, and 4 food groups or nutrients to discourage: refined grains, sodium, added sugars, and saturated fat (32).

### The NRF Family of Nutrient Density Scores

The Nutrient Rich Foods (NRF) models are typically based on two subscores: NR and LIM. NRn is based on a variable number n of nutrients to encourage whereas LIM is based on the same 3 nutrients to limit (saturated fat, added sugars and sodium) (10, 11, 23). In prior studies, the number of nutrients to encourage has varied from 3 to 23 (11, 12, 23). Reference daily values (DVs) were based on the US Food and Drug Administration (FDA) values and on other standards. The original NRF9.3 score (34) was calculated as follows:

$$\begin{array}{l} NRFn.3=\left(NRn-LIM\right), \end{array}$$

Where NR and LIM are given by:

$$\begin{array}{lll} NR & = & \overset{n}{\underset{i=1}{\sum }}\frac{\frac{Intake_{i}}{Energy}\times 100,}{DV_{i}}\\ LIM & = & \overset{n}{\underset{i=1}{\sum }}\frac{\frac{Intake_{i}}{Energy}\times 100}{DV_{i}}-1. \end{array}$$

In this formulation, intake_i_ was the daily intake of each nutrient i and DVi is the reference daily value for that nutrient, expressed in percentage of DV per 100 kcal. Maximum recommended values (MRVs) were used for nutrients to limit. As in past calculations, percent DVs for nutrients were truncated at 100%.

The new hybrid NRF score for individual foods was calculated as:

$$\begin{array}{l} \text{NRFh }=\;100^{*}\left(\text{NRx }+\text{ MPy }-\text{ LIMz}\right), \end{array}$$

where the new element MP stands for as yet to be determined number of My Plate food groups to encourage. Thus:

$$\begin{array}{lll} \text{NRx}\left(\text{or x qualifying nutrients to encourage}\right) & = & \overset{x}{\underset{i=1}{\sum }}\frac{{Nutrient/energy\;density}_{i}}{DV_{i}},\\ \text{MPy}\left(\text{y qualifying food groups to encourage}\right) & = & \overset{y}{\underset{i=1}{\sum }}\frac{{Food\;Group}_{i}}{{DGA\;Recommendation}_{i}},\\ \text{LIMz}\left(\text{z disqualifying nutrients to limit}\right) & = & \overset{z}{\underset{i=1}{\sum }}\frac{{Nutrient/energy\;density}_{i}}{MRV_{i}}. \end{array}$$

In past studies, diets assessed using various NRF scores were compared to an independent measure of a healthy diet, the HEI 2005 score, using multiple regressions. The goal of present analyses was to test which combinations of nutrients to encourage (or to limit) along with selected food groups gave a total score that would account for most of the variance when HEI-2015 is regressed on the nutrients/food group intakes.

Following similar procedures, regression analyses were used to assess whether including one or all of the candidate nutrients and food groups would improve the ability of the new nutrient density score to predict HEI (35). Three sets of models were tested: (1) nutrients only; (2) food groups only; and (3) both nutrients and food groups.

### Nutrient and Food Group Standards

Daily values for nutrients used in developing and testing the new NP models are summarized in Table 1. The recommended daily values for MP food groups, based on the 2015–2020 DGA, are expressed in cup equivalents or ounce equivalents per day. The values were set as follows: whole grain 3 oz.eq; vegetables 2.5 cup eq; fruit 2 cup eq; dairy 3 cup eq; and nuts and seeds 0.7 oz.eq. These values were based on a 2,000 calorie US-Style Healthy Eating Pattern.

**Table 1:** Daily values for nutrients and MyPyramid food groups used in developing and testing new NP model.

**Nutrient**	**DV**
**NR NUTRIENTS**	
Protein (g)	50
Dietary fiber (g)	28
Vitamin A, RAE (mcg)	900
Vitamin B12 (mcg)	2.4
Vitamin C (mg)	90
Vitamin D (D2 + D3) (μg)	20
Vitamin E as alpha-tocopherol (mg)	15
Folate, DFE (mcg)	400
Calcium (mg)	1,300
Iron (mg)	18
Potassium (mg)	4,700
Magnesium (mg)	420
Mono- and Poly-unsaturated fat (g)	58
**LIM NUTRIENTS**	
Total saturated fatty acids (g)	20
Sodium (mg)	2,300
Added sugars (g)	50
**Recommended My Plate food groups**	Servings/day (per 2,000 kcal)
Total dairy (cup eq)	3
Whole grain (oz eq)	3
Nuts and seeds (oz eq)	0.7
Total fruits (cup eq)	2
Total vegetables (w/o legumes (cup eq)	2.5

### Analytical Procedures

Analyses used SAS 9.4 (SAS Institute, Cary NC) with survey parameters including strata, primary sampling units and day 1 dietary subsample weights. For each subject, the independent variables were intakes of a given nutrient or food group, calculated per 100 kcal as a proportion of daily value and capped at 1.0. For each nutrient or MP food group the formula was 100^*^ (total daily nutrient _i_/total daily calories)/DV_i_., where DV_i_ is the daily value for each nutrient i.

Individual nutrient and MP food group scores were determined and related to HEI-2015 diet quality scores using max r-square regression analysis. The 21 variables were split into 16 nutrients and 5 food groups. The maximum r-square was developed using proc surveyreg with sampling weights, strata and primary sampling units for each set of analyses (nutrients only, food groups only, and both nutrients and food groups). For the combined set of 21 nutrient/food group independent variables there were 2^*^21 = 2,097,152 possible models using all subsets of these 21 variables. All 2,097,152 regressions are performed and r-square calculated. The results table shows for each number of variables in the model, the one resulting in the maximum r-square. Effectively, this was a weighted NRF with weights being the regression coefficients for each component (34).

The models were tested within subpopulations of interest. Within each subpopulation, HEI-2015 total scores were regressed on the predicted values to obtain a beta and r-square. This was done to test how well the NRF scores, which had been calculated from the total population predicted HEI-2015 values within subpopulations. The r-squares for the subpopulation regression analyses were compared to the r-square for the total population.

## Results

### Regression Models to Identify Index Nutrients and MP Food Groups

Table 2 shows the results of regression models for 16 nutrients including candidate nutrients to encourage and nutrients to limit. The NRF nutrient density scores were related to HEI-2015 by r-square when regressed on different subsets of the nutrients. The maximum R^2^ model is shown for each number of independent variables in the model. No covariates were used in the models.

**Table 2:** Regression models for nutrients to encourage and nutrients to limit (n = 16).

	**NRF5**	**NRF6**	**NRF7**	**NRF8**	**NRF9**	**NRF10**	**NRF12**	**NRF15**	**NRF16**
**NUTRIENT**									
Potassium	4.90	4.19	3.43	2.54	2.32	2.28	1.84	1.76	1.80
Dietary fiber	3.04	2.60	2.85	2.43	2.64	2.66	2.76	2.84	2.85
PUFA/MUFA	3.04	2.62	2.54	2.34	2.55	2.55	2.61	2.71	2.70
Vitamin D			1.37	1.21	1.38	1.15	1.09	0.93	0.93
Magnesium				1.52	1.66	1.44	1.33	1.42	1.42
Calcium						0.54	0.54	0.54	0.55
Vitamin C							0.11	0.12	0.13
Protein							0.30	0.25	0.25
Vitamin B12								0.08	0.09
Sat fat	−2.25	−2.47	−2.56	−2.45	−2.48	−2.66	−2.64	−2.68	−2.67
Sodium	−1.57	−1.82	−1.77	−1.75	−1.74	−1.76	−1.85	−1.84	−1.84
Added sugar		−9.96	−9.47	−8.69	−9.19	−9.03	−8.31	−8.42	−8.40
Folate, DFE					−0.30	−0.32	−0.32	−0.31	−0.31
Vitamin E								−0.14	−0.12
Iron								−0.16	−0.14
Vitamin A									−0.05
***R***^**2**^	**0.59**	**0.62**	**0.64**	**0.64**	**0.65**	**0.65**	**0.66**	**0.66**	**0.66**

Table 2 clearly identifies the principal nutrients to encourage and the nutrients to limit. The principal nutrients to encourage were potassium, fiber, dietary PUFA+MUFA, magnesium and calcium, followed by vitamin C, protein, and vitamin B12. Nutrients to limit were identified as saturated fat, sodium and added sugar, consistent with all past NRF and other NP models (35). The r-square values reached an asymptote around NRF9 or NRF10 of about 65%; adding additional elements to the model at that point did not explain additional variance. The NRF 16 model explained 66% of the variance.

Table 3 shows the results of regression models for 5 MP food groups. The MP models were related to HEI-2015 by r-square when HEI-2015 was regressed on different subsets of MP food groups. Table 3 shows that the strongest results were obtained for whole grains, total fruit, nuts and seeds, followed by vegetables and total dairy. The MP4 and the MP5 models each accounted for 50% of the variance in HEI-2015 total scores.

**Table 3:** Regression models for MyPyramid food groups encourage (five food groups).

	**MP1**	**MP2**	**MP3**	**MP4**	**MP5**
**Food group**					
Whole grains	2.60	2.35	2.21	2.17	2.16
Total fruit	1.50	1.47	1.47	1.41	1.40
Nuts seeds			0.41	0.41	0.41
Vegetables				1.11	1.14
Total dairy					0.23
***R***^**2**^	**0.19**	**0.35**	**0.45**	**0.50**	**0.50**

Table 4 shows the results of regression models for both 16 nutrients and for 5 MP food groups combined. The HEI-2015 was regressed on different subsets of nutrients and MP food groups. The principal nutrients to encourage were potassium, fiber, and dietary PUFA+MUFA, followed by vitamin D and protein. The principal MP food groups were whole grains, fruit, and dairy. Nutrients to limit were clearly identified again as saturated fat, sodium, and added sugar. A combined NRF 10 model explained 72% of variance in HEI-2015.

**Table 4:** Regression models for 16 nutrients and for five MyPyramid food groups.

	**NRF5**	**NRF6**	**NRF7**	**NRF8**	**NRF9**	**NRF10**	**NRF12**	**NRF15**	**NRF16**
**Nutrient**									
Potassium	6.56	5.50	4.67	4.43	4.05	3.42	3.01	2.38	1.97
**Whole grains**	2.01	1.86	1.86	1.80	1.77	1.54	1.50	1.60	1.52
PUFA/MUFA	3.30	2.67	3.00	2.23	2.83	2.94	2.71	2.73	2.75
**Fruit**			0.65	0.64	0.69	0.60	0.57	0.68	0.71
**Dairy**					0.85	0.93	0.77	1.18	1.30
Dietary fiber						1.20	1.53	1.54	1.42
**Nuts seeds**				0.20	0.19	0.16	0.17	0.17	0.15
Vit D							0.82	0.72	0.79
Protein							0.44	0.42	0.37
**Vegetables**								0.26	0.29
Magnesium									0.61
Sat fat	−2.40	−2.59	−2.47	−2.31	−2.77	−2.72	−2.72	−2.66	−2.65
Sodium	−1.53	−1.80	−1.58	−1.40	−1.33	−1.36	−1.34	−1.45	−1.45
Added sugar		−10.62	−8.96	−8.79	−8.02	−7.07	−7.51	−6.36	−6.38
***R***^**2**^	**0.62**	**0.66**	**0.68**	**0.70**	**0.71**	**0.72**	**0.73**	**0.74**	**0.74**

*Total number of NRF elements = 21. Shown are maximum r square beta coefficients for each model and the overall regression R square*.

### Candidate Hybrid NRFh Models

Based on data in Table 4, we identified two NRF models for further testing. The overall model structure followed the NRF framework:

$$\begin{array}{l} \text{NRFh }=\;100\;*\;\left(\text{NRx }+\text{MPz}-\text{ LIMy}\right). \end{array}$$

The first model NRFh-3.3.4 was composed of three nutrients to encourage (fiber, potassium, PUFA+MUFA), three nutrients to limit (saturated fat, sodium, added sugar) and four MP groups to encourage (whole grains, dairy, fruit, and nuts and seeds). The second model NRFh-4.3.3 was based on four nutrients to encourage (potassium, fiber, dietary MUFA+PUFA, and protein), three nutrients to limit (saturated fat, sodium, and added sugar) and three MP food groups to encourage (whole grains, dairy, and fruit).

Table 5 shows the results of regression models for each NRFh model components and for the total NRFh model. The two models were very similar and each NRF model accounted for 72% of the variance. Table 5 shows the association of each component with HEI 2015 with regression coefficient helping to discern direction and magnitude of association (though need to factor in metric used, e.g., g vs. cup equivalent).

**Table 5:** The two models: NRFh3.4.3 and 2 NRFh4.3.3.

**NRFh**	**Factor**	**Beta**	**SE**	**P**
NRFh3.4.3				
	Intercept	47.37	1.17	<0.0001
**3**:4:3	Fiber	1.20	0.10	<0.0001
**3**:4:3	Potassium	3.42	0.32	<0.0001
**3**:4:3	MUFA+PUFA	2.94	0.09	<0.0001
3:**4**:3	Dairy_total	0.93	0.05	<0.0001
3:**4**:3	Fruit_total	0.60	0.05	<0.0001
3:**4**:3	Whole grains	1.54	0.06	<0.0001
3:**4**:3	Nuts, seeds	0.16	0.01	<0.0001
3:4:**3**	Added sugar	−0.44	0.02	<0.0001
3:4:**3**	Saturated fat	−2.72	0.06	<0.0001
3:4:**3**	Sodium	−1.36	0.08	<0.0001
			***R***^**2**^	**0.72178**
NRFh4.3.3				
	Intercept	43.31	1.14	<0.0001
**4**:3:3	Protein	0.50	0.06	<0.0001
**4**:3:3	Fiber	1.63	0.11	<0.0001
**4**:3:3	Potassium	2.93	0.35	<0.0001
**4**:3:3	MUFA+PUFA	3.65	0.09	<0.0001
4:**3**:3	Dairy_total	0.95	0.05	<0.0001
4:**3**:3	Fruit_total	0.65	0.05	<0.0001
4:**3**:3	Whole grains	1.52	0.06	<0.0001
4:3:**3**	Added sugar	−0.35	0.02	<0.0001
4:3:**3**	Saturated fat	−2.83	0.06	<0.0001
4:3:**3**	Sodium	−1.65	0.12	<0.0001
			***R***^**2**^	**0.71592**

*Shown are maximum r square beta coefficients for each model component and the overall regression R square. NRFh model codes corresponding to nutrients or food groups are indicated in bold type*.

### NRFh4.3.3 and NRFh3.3.4 Models by Demographics

**Figure 1:**
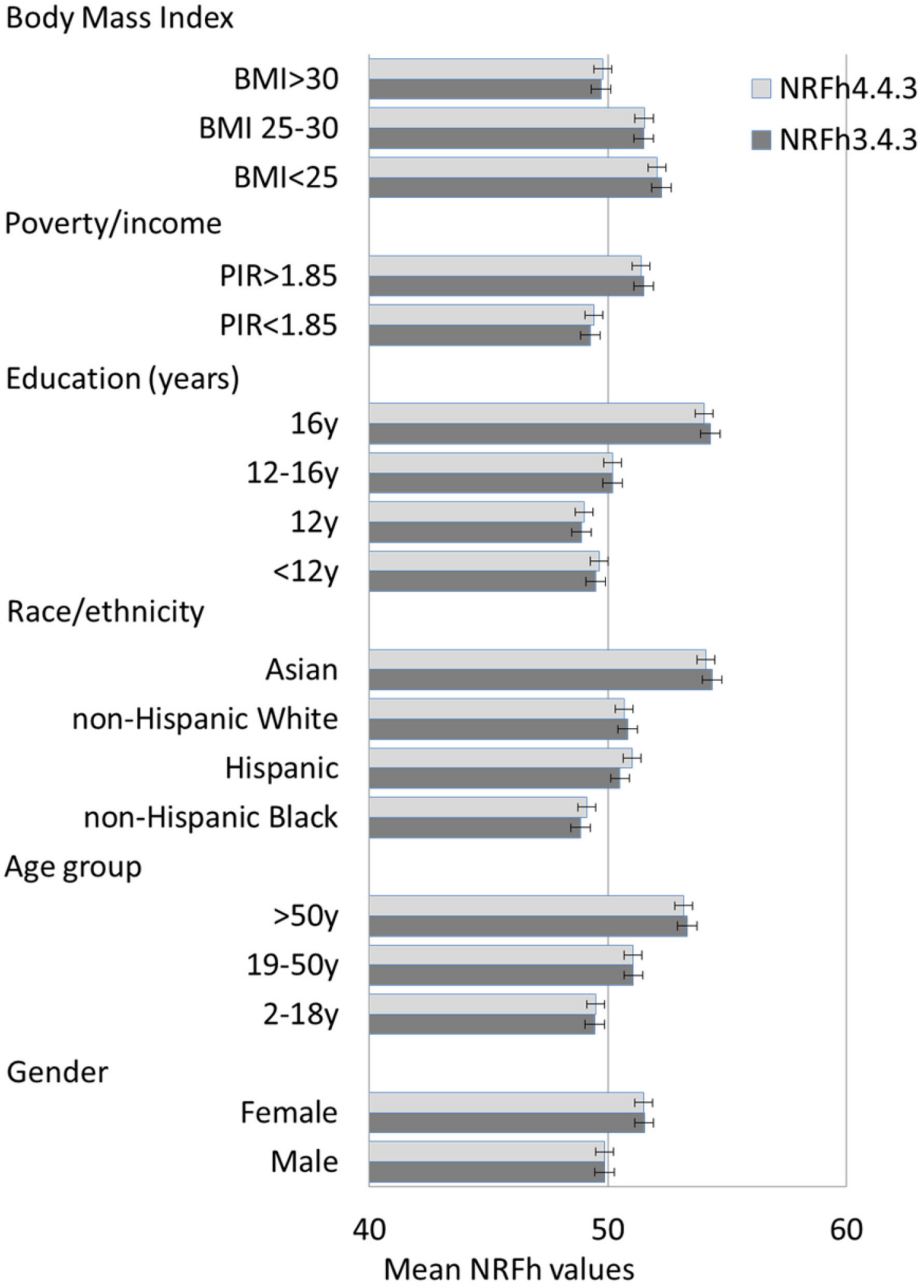
Mean NRFh values (and SEM) by model type and socio-demographics of the NHANES population sample.

Figure 1 shows mean NRFh values (and SEM) for the two models by sociodemographic variables. The results were consistent with the known distribution of HEI across population subgroups. Dietary nutrient density was influenced by gender, age group, race/ethnicity, education, incomes, and obesity status. Higher NRFh nutrient density scores were obtained for women, older adults, Asians, individuals with higher education and higher incomes, and for persons with BMI below 30. The two models NRFh.3.4.3 andNRFh.4.3.3 were identical. When it came to regressions, each model accounted for about 70% of the variance for all population subgroups. Slightly lower r-square values were obtained for Asians and for persons with obesity. The results of regressions, beta coefficients and R^2^ values are shown in Supplementary Table 1.

## Discussion

The goal of NP models is to capture nutrient density of foods. Such models are very often nutrient based. Not only that, they are based mostly on nutrients to limit—fat, saturated fat, total or added sugars, and sodium. As a result, negative nutrient density scores are highly correlated with energy density and do not capture the full nutritional value of foods.

The current dietary guidelines continue to stress the importance of limiting calories, saturated fat, added (or free) sugar, and sodium. However, the DGA also feature healthy food patterns and healthy food ingredients. NP models have not yet followed suit. Some models (notably FSA Ofcom) add an arbitrary number of points to foods containing FVN (fruit, vegetables, nuts). The FSA-Ofcom and the Australian Health Star Rating scores award extra points for foods content of fruit vegetables and nuts (15, 18). The French NutriScore features vegetables, fruit and nuts, whereas SENS lists fruit, vegetables, legumes and nuts (36). However, most of these NP models are heavily weighted by nutrients to limit and (sugar and fat) and some (FSA –Ofcom) capture food energy density rather than food nutrient content. This was pointed out as far back as 2008 (23).

The present goal was to identify a nutrient profile model that considered both nutrients and food groups and determine which combination of elements would best be associated with highest-quality diets. We took the basic NRF format where NRF = NR + MP -LIM. Then we used regression analyses to look for correspondence between alternative NRF mixed models and HEI 2015 scores. Calculations are best based on 100 kcal of food, to avoid conflating energy density and nutrient density. First, regressions based on nutrients only (starting with 16 nutrients) showed a R^2^ of ~65%. Regressions based on five food groups had a R^2^ of about 50%. Mixed models put 16 nutrients and five food groups into a regression and provided higher r R^2^ values than either the nutrient alone or the food group alone models.

Two best fitting models—called NRFh-3.4.3 and NRFh-4:3:3 incorporated nutrients and MP food groups. Nutrients to encourage were fiber, potassium, PUFA+MUFA—with or without protein. The new MP food groups were dairy, fruit, whole grains with, or without nuts/seeds. The LIM component was the same—saturated fat, added sugar and sodium. Both models accounted for 72% of the variance. Regressions were significant for each socio-demographic population subgroup evaluated.

The DGA are moving toward healthy food patterns—the 2015 DGA did that and it appears 2020 DGA will do the same. For example, food groups considered to be nutrient-dense or nutrient-rich typically include whole grains, low fat milk and dairy, fruits, vegetables, lean meats, poultry and fish, eggs, beans and peas, and nuts and seeds, all prepared without or minimal added fats, sugars, or sodium. Many of those foods scored high on the Nutrient Rich Foods (NRF) index.

The present goal was to align NP models with the DGA. Regression analyses showed that the candidate food groups include whole grains, dairy, fruit and possibly nuts, but interestingly not vegetables. This is consistent with the argument that dairy and fruit add more to NRF scores than do FVN. One more problem: calculating FVN content can be complex. Public Health England document says that the calculation methods were intended for manufacturers, not the general public. An alternative approach is to use information provided on the back of pack ingredient panel. Dairy and fruit listed as first ingredients were associated with higher NRF8.3 scores (26). However, developing branded databases of quantitative food group information as well as incorporating detailed data on food group intake in national dietary surveys should be undertaken to provide a better estimate of food groups intakes, the contribution of specific foods to recommended food groups and to aid with food group claims modernization, such as the potential to updated FDA Healthy claim to include food groups (26).

Given the shift in DGA, NP methods need to innovate to keep pace with the latest trends. First, NP models need to incorporate the recommended food groups (MyPlate) as the current study begins to address. Second, future DGA might benefit from formal metrics of nutrient density, allowing a quantitative comparison among alternative healthy food patterns.

## Conclusion

Two new hybrid scores NRFh-3.4.3 and NRFh-4.3.3 using regression analyses accounted for 72% of the variance in total HEI-2015 total scores. The scores were based on nutrients to encourage, MP food groups to encourage, and nutrients to limit. The novel component of MP food groups to encourage included dairy, fruit, and whole grains. Nutrient profiling models that include both nutrients and food groups align better with the current Dietary Guidelines for Americans. Food-oriented data bases such as FPED, when developed globally, will aid in the development of food based dietary guidance.

## Data Availability Statement

All data used in this study were obtained from publicly available NHANES or USDA websites. Those that need help in assembling these datafiles together to replicate our analyses should contact Victor L. Fulgoni, vic3rd@aol.com.

## Author Contributions

AD and VF conceptualized the study. VF conducted the analyses. AD took the lead on preparing the manuscript. Both authors revised and reviewed the manuscript and approved it for publication and agree to be accountable for the content of the work.

## Conflict of Interest

AD has received grants, contracts, honoraria, and consulting fees from numerous food and beverage companies and other commercial and non-profit entities with interests in nutrient profiling, diet quality and health. VF was Senior Vice-President of Nutrition Impact and provides food and nutrition consulting services for food and beverage companies.
