# Supplementary material for: New Nutrient Rich Food Nutrient Density Models That Include Nutrients and MyPlate Food Groups
Source: Front Nutr. 2020 Jul 21;7:107. doi: 10.3389/fnut.2020.00107 (PMC7387572; doi:10.3389/fnut.2020.00107)
Supplement: Supplementary file 1 [file Table_1.docx]

**Drewnowski A, Fulgoni V IIIrd. New Nutrient Rich Food nutrient density models that include nutrients and MyPlate food groups**

**Supplemental Table 1.** The two NRF models by demographics. Statistics are for regression to HEI for each population subgroup

| **Subpopulation** | **N** | **Model 1 NRF 3:4:3** | | | | | | **Model 2 Score 4:3:3** | | | | | |
| --- | --- | --- | --- | --- | --- | --- | --- | --- | --- | --- | --- | --- | --- |
|  |  | **Mean** | **SE** | **Beta** | **SE** | **P** | **Rsquare** | **Mean** | **SE** | **Beta** | **SE** | **P** | **Rsquare** |
| All | 23,643 | 50.696 | 0.199 | 1.000 | 0.011 | 0 | 0.72178 | 50.696 | 0.190 | 1.000 | 0.013 | 0 | 0.71592 |
| Gender |  |  |  |  |  |  |  |  |  |  |  |  |  |
| Male | 11,790 | 49.857 | 0.192 | 1.025 | 0.017 | 0 | 0.72503 | 49.875 | 0.192 | 1.033 | 0.017 | 0 | 0.72283 |
| Female | 11,853 | 51.528 | 0.258 | 0.979 | 0.014 | 0 | 0.71754 | 51.510 | 0.246 | 0.972 | 0.016 | 0 | 0.70876 |
| Age group |  |  |  |  |  |  |  |  |  |  |  |  |  |
| 2-18y | 8,654 | 49.443 | 0.224 | 1.080 | 0.016 | <0.0001 | 0.72618 | 49.495 | 0.229 | 1.067 | 0.015 | <0.0001 | 0.72480 |
| 19-50 y | 14,989 | 51.077 | 0.222 | 0.979 | 0.013 | <0.0001 | 0.72130 | 51.061 | 0.210 | 0.981 | 0.014 | <0.0001 | 0.71410 |
| > 50y | 6,944 | 53.342 | 0.312 | 0.934 | 0.021 | <0.0001 | 0.70336 | 53.184 | 0.301 | 0.943 | 0.023 | <0.0001 | 0.69925 |
| Ethnicity |  |  |  |  |  |  |  |  |  |  |  |  |  |
| Hispanic | 6,481 | 50.517 | 0.235 | 1.078 | 0.014 | <0.0001 | 0.73178 | 51.011 | 0.234 | 1.061 | 0.015 | <0.0001 | 0.73389 |
| non Hisp White | 8,024 | 50.840 | 0.266 | 0.993 | 0.016 | <0.0001 | 0.72528 | 50.685 | 0.265 | 0.997 | 0.018 | <0.0001 | 0.71816 |
| non Hisp Black | 5,627 | 48.864 | 0.277 | 0.970 | 0.016 | <0.0001 | 0.70348 | 49.110 | 0.278 | 0.971 | 0.016 | <0.0001 | 0.70442 |
| Asian | 2,484 | 54.378 | 0.504 | 0.894 | 0.019 | <0.0001 | 0.67832 | 54.108 | 0.525 | 0.877 | 0.020 | <0.0001 | 0.66553 |
| Poverty/income |  |  |  |  |  |  |  |  |  |  |  |  |  |
| PIR <= 1.85 | 11,245 | 49.249 | 0.238 | 0.980 | 0.012 | <0.0001 | 0.70683 | 49.412 | 0.234 | 0.973 | 0.012 | <0.0001 | 0.70435 |
| PIR > 1.85 | 10,552 | 51.500 | 0.253 | 1.005 | 0.017 | <0.0001 | 0.72681 | 51.378 | 0.250 | 1.009 | 0.019 | <0.0001 | 0.72063 |
| PIR missing | 1,846 | 51.423 | 0.556 | 0.996 | 0.032 | <0.0001 | 0.71607 | 51.635 | 0.562 | 0.992 | 0.036 | <0.0001 | 0.70915 |
| Education |  |  |  |  |  |  |  |  |  |  |  |  |  |
| HS | 11,725 | 49.504 | 0.184 | 1.026 | 0.014 | <0.0001 | 0.70556 | 49.641 | 0.187 | 1.009 | 0.013 | <0.0001 | 0.70099 |
| HS | 3,550 | 48.903 | 0.353 | 0.937 | 0.025 | <0.0001 | 0.69527 | 48.993 | 0.359 | 0.919 | 0.032 | <0.0001 | 0.68324 |
| Some College | 4,664 | 50.235 | 0.330 | 1.009 | 0.016 | <0.0001 | 0.73581 | 50.217 | 0.312 | 1.009 | 0.017 | <0.0001 | 0.72938 |
| College+ | 3,692 | 54.287 | 0.342 | 0.954 | 0.029 | <0.0001 | 0.71718 | 54.037 | 0.325 | 0.979 | 0.029 | <0.0001 | 0.71932 |
| Adult BMI |  |  |  |  |  |  |  |  |  |  |  |  |  |
| < 25 | 4,419 | 52.249 | 0.364 | 0.992 | 0.019 | <0.0001 | 0.74170 | 52.065 | 0.347 | 0.999 | 0.019 | <0.0001 | 0.73294 |
| 25-30 | 4,810 | 51.519 | 0.306 | 0.979 | 0.021 | <0.0001 | 0.72895 | 51.525 | 0.313 | 0.971 | 0.027 | <0.0001 | 0.71844 |
| > 30 | 5,604 | 49.684 | 0.224 | 0.952 | 0.024 | <0.0001 | 0.68881 | 49.785 | 0.221 | 0.960 | 0.024 | <0.0001 | 0.68843 |
| Child BMI-z |  |  |  |  |  |  |  |  |  |  |  |  |  |
| Percentile < 85 | 5,588 | 49.505 | 0.279 | 1.086 | 0.017 | <0.0001 | 0.73595 | 49.500 | 0.286 | 1.074 | 0.017 | <0.0001 | 0.73201 |
| Percentile 85-95 | 1,372 | 48.975 | 0.422 | 1.078 | 0.027 | <0.0001 | 0.70449 | 49.107 | 0.428 | 1.056 | 0.027 | <0.0001 | 0.70606 |
| Percentile > 95 | 1,571 | 49.364 | 0.456 | 1.057 | 0.055 | <0.0001 | 0.70611 | 49.575 | 0.477 | 1.044 | 0.049 | <0.0001 | 0.71308 |
|  |  |  |  |  |  |  |  |  |  |  |  |  |  |
